# New insights into the ferroptosis and immune infiltration in endometriosis: a bioinformatics-based analysis

**DOI:** 10.3389/fimmu.2024.1507083

**Published:** 2025-01-13

**Authors:** Lusha Liu, Feifei Han, Naiyi Du, Yakun Liu, Aihong Duan, Shan Kang, Bin Li

**Affiliations:** ^1^ Department of Gynecology, The Fourth Hospital of Hebei Medical University, Shijiazhuang, China; ^2^ Department of Gynecology, Handan Central Hospital, Handan, China

**Keywords:** ferroptosis, endometriosis, WGCNA, immune infiltration, immune checkpoint genes

## Abstract

**Background:**

Ferroptosis, a recently discovered iron-dependent cell death, is linked to various diseases but its role in endometriosis is still not fully understood.

**Methods:**

In this study, we integrated microarray data of endometriosis from the GEO database and ferroptosis-related genes (FRGs) from the FerrDb database to further investigate the regulation of ferroptosis in endometriosis and its impact on the immune microenvironment. WGCNA identified ferroptosis-related modules, annotated by GO & KEGG. MNC algorithm pinpointed hub FRGs. Cytoscape construct a ceRNA network, and ROC curves evaluated diagnostic efficacy of hub FRGs. Consensus cluster analysis identified ferroptosis subclusters, and CIBERSORT assessed immune infiltration of these subclusters. Finally, RT-qPCR validated hub FRG expression in clinical tissues.

**Results:**

We identified two ferroptosis modules of endometriosis, and by enrichment analysis, they are closely linked with autophagy, mTOR, oxidative stress, and FOXO pathways. Furthermore, we identified 10 hub FRGs, and the ROC curve showed better predictive ability for diagnosing. RT-qPCR confirmed that the tissue expression of 10 hub FRGs was mostly consistent with the database results. Subsequently, we developed a ceRNA network based on 4 FRGs (BECN1, OSBPL9, TGFBR1, GSK3B). Next, we identified two ferroptosis subclusters of endometriosis and discovered that they are closely linked with endometriosis stage. Importantly, immune enrichment analysis illustrated that the expression levels of immune cells and immune checkpoint genes were significantly different in the two ferroptosis subclusters. Specifically, the ferroptosis subcluster with stage III-IV of endometriosis is more inclined to the immunosuppressive microenvironment.

**Conclusions:**

Our study showed that ferroptosis may jointly promote endometriosis progression by remodeling the immune microenvironment, offering new insights into pathogenesis and therapeutics.

## 1 Introduction

Endometriosis (EMs), an estrogen-dependent inflammatory disease, is characterized by abnormal growth of endometrial-like tissues outside the uterus, affecting approximately 10% of women of reproductive age globally (1). Common symptoms include pelvic pain and infertility, which severely jeopardize the physical and mental health of patients (2). Hormone drugs and surgery are the primary treatments for EMs; however, these treatments are closely linked to various adverse effects, such as contraception and menopause-related symptoms (1). Currently, the etiology of EMs remains elusive. Previous studies have shown that iron overload and excessive accumulation of reactive oxygen species (ROS) are pathological characteristics of EMs (3, 4), which lays the foundation for the occurrence of cellular ferroptosis.

Ferroptosis, a novel form of iron-dependent programmed cell death, is characterized by the accumulation of ROS and lipid peroxidation-mediated membrane damage (5). Mechanistically, ferroptosis stands apart from other forms of cell death, such as apoptosis, autophagy, and necrosis, primarily due to distinct differences observed in cell morphology, metabolism, and protein expression patterns (5). Ferroptotic cell death manifests as damage of the mitochondrial membrane, increased membrane density, reduced mitochondrial cristae, and intact nuclear membranes (5). The critical mechanisms underlying ferroptosis are intricately linked to iron metabolism, lipid metabolism, and glutathione metabolism (6). Several lines of study showed that ferroptosis played a crucial role in the pathogenesis of various diseases, including EMs (7–10). Prior investigations have elucidated the complex function of ferroptosis in EMs, highlighting its significance in disease development and progression (11–13). On the one hand, endometriotic lesions present resistance to ferroptosis, impeding the clearance of ectopic endometrium and facilitating its proliferation and migration (11, 14), while ferroptotic cell death also release inflammatory cytokines and trigger downstream regulatory pathways, thereby promoting proliferation and angiogenesis in adjacent tissues (15). However, the exact mechanisms of ferroptosis in EMs remains poorly understood and needs further investigation.

As we all know, immune dysfunction, characterized by abnormalities in the number and function of immune cells, stands as a pivotal feature of EMs (16). Recent studies have highlighted the close relationship between ferroptosis and the immune microenvironment as well as immunotherapy for diseases (5). For instance, IL-6 from M1 macrophages can induce lipid peroxidation and disrupt iron homeostasis in bronchial epithelial cells, leading to ferroptosis (17). Ferroptotic cell death could also activate the innate immune system and promote the development of inflammatory diseases by releasing the damage-associated molecular patterns (DAMPs). Additionally, ferroptotic cell death exposes tumor antigens, boosting tumor cell immunogenicity and enhancing the effectiveness of immunotherapy (18). Therefore, the exploration of the interaction between ferroptosis and immune cell infiltration in EMs is crucial for understanding the pathogenesis and developing effective treatment strategies of the disease.

In this study, we initially employed the weighted gene co-expression network analysis (WGCNA) to identify ferroptosis-related modules in EMs samples and annotated their potential functions. Subsequently, we constructed subclusters of EMs based on ferroptosis-related genes (FRGs), and evaluated the immune infiltration of subclusters. Following this, we identified hub FRGs and constructed a ceRNA network for hub FRGs, and further validated their expression levels in tissue samples through RT-qPCR. Taken together, our study reveals new insights into the mechanism of ferroptosis in the development of EMs.

## 2 Methods

### 2.1 Data acquisition

The microarray data of EMs, including mRNA (GSE51981), miRNA (GSE105765), and lncRNA (GSE105764), were obtained from GEO databases (19). FRGs were acquired from the FerrDb database (20).

### 2.2 Weighted gene coexpression network analysis

We used the ‘WGCNA’ package to reconstruct coexpression gene networks associated with disease clinical characteristics in GSE51981. Subsequently, we determined an appropriate soft threshold power and employed a dynamic tree-cut strategy to hierarchically cluster and merge similar modules. Next, we calculated the module eigengene, module membership, and gene significance to identify clinical trait modules.

### 2.3 Functional annotation of key modules and identification of hub FRGs

Gene Ontology (GO) biological process and Kyoto Encyclopedia of Genes and Genomes (KEGG) pathway enrichment analyses were performed using the clusterProfiler package to further interpret and visualize diverse biological functions of those key modules. Then, WGCNA was firstly utilized to identify gene−gene interactions in two important modules, and then the hub FRGs were obtained using the MNC genetic algorithm. To further confirm the accuracy of the results, we validated the expression of these hub FRGs in clinical tissue samples. Moreover, the receiver operating characteristic (ROC) analysis was performed to assess the specificity and sensitivity of these hub FRGs for diagnosing EMs.

Identification of new ferroptosis subclusters of endometriosis and immune infiltration analysis

We analyzed the expression of ferroptosis genes in endometriosis samples of GSE51981 using consensus cluster analysis to identify novel ferroptosis molecular subclusters. Subsequently, the CIBERSORT package was utilized to estimate the relative expression percentages of 22 infiltrated immune cell types for each subcluster. Meanwhile, the expression levels of immune checkpoint genes in the subclusters were assessed using Wilcoxon tests.

### 2.4 Construction of a ceRNA network

TargetScan (21), miRDB (22), and miRTarBase (23) were utilized for miRNA prediction, while Mircode was employed for predicting miRNA−lncRNA interactions with a stringent approach (24). Subsequently, a lncRNA−miRNA interaction network was established (**Additional File 1**). DESeq2 R package was then applied to analyze the differential expression of lncRNAs (**Additional File 2**), and lncRNAs with a significance level of *P*<10^-6^ were chosen to construct the ceRNA network (**Additional File 3**). The interaction networks were visualized using Cytoscape software.

### 2.5 Collection of tissue samples

In this study, based on the use of specimens in the GSE51981 dataset, we also used eutopic endometrium from ovarian endometriosis (OEM) patients (n=22) as case group, which allowed us to investigate the molecular changes in the endometrium associated with the disease process. Furthermore, we used normal endometrium from cervical intraepithelial neoplasia grade III (CIN III) patients (n=10) as a control group. The comparison between these two types of samples highlights the differences in gene expression between control and diseased endometrium. All samples were obtained from the Department of Gynecology at the Fourth Hospital of Hebei Medical University in China. Patients who underwent hormone therapy within six months prior to surgery or exhibited concurrent tumor conditions were excluded from the study. This research was approved by the Ethics Commission of the Fourth Affiliated Hospital of Hebei Medical University (2021KY016). Written informed consent was obtained from all patients.

### 2.6 Total RNA extraction and RT−qPCR

TRIzol reagent (Thermo Fisher Scientific, Waltham, USA) was utilized for total RNA extraction from the tissues, with RNA quality assessed using a Nanodrop 2000 spectrophotometer (Thermo Fisher Scientific Inc., Waltham, MA, USA). Subsequently, 1μg of total RNA was reverse transcribed into cDNA employing SweScript All-in-One First-Strand cDNA Synthesis SuperMix (Servicebio, Wuhan, China), followed by qPCR using Hieff^®^ qPCR SYBR Green Master Mix (YEASEN, Shanghai, China). The PCR procedure as follows: 95°C for 5 min followed by 40 cycles at 95°C for 10 s, 58°C for 20 s, and 72°C for 20 s. Primer sequences of genes can be found in **Additional File 4**. Each sample underwent triplicate analysis, with gene expression levels normalized to GAPDH and calculated using the 2^- ΔΔCT^ method.

### 2.7 Statistical analysis

GraphPad software version 8.0 was utilized for statistical analyses. An unpaired t-test was conducted to ascertain the statistical significance of pairwise differences between groups, with a significance level set at *P*<0.05. The diagnostic efficacy of the hub FRGs was assessed through ROC curve analysis.

## 4 Results

### 4.1 Identification of key modules

**Figure 1:**
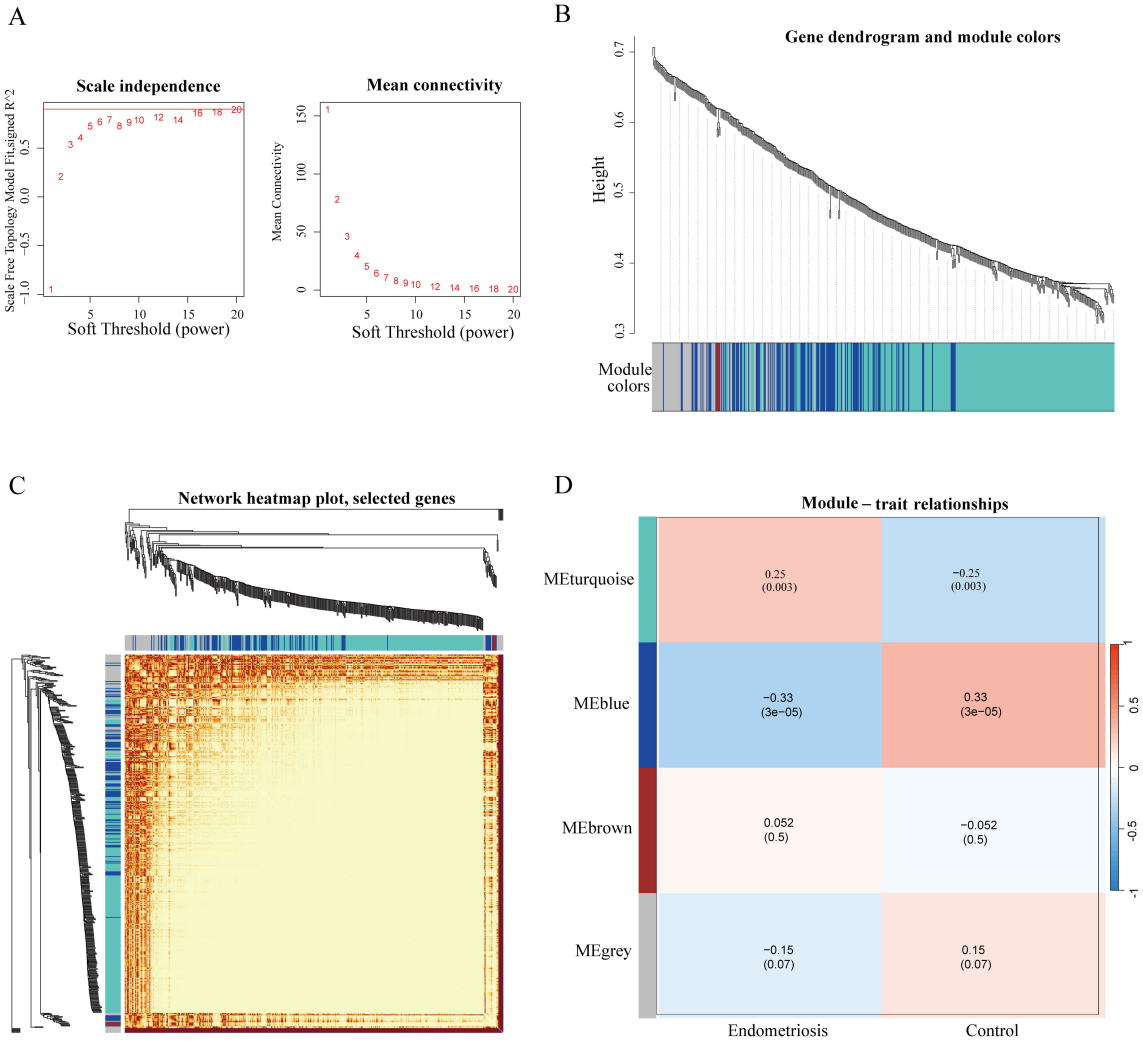
Identification of ferroptosis-related modules of endometriosis by WGCNA analysis. **(A)** Network topology analysis for various soft threshold powers (The association between the scale-free fit index and diverse soft-thresholding exponents (former); The connection between average connectivity and diverse soft-thresholding exponents (latter). **(B)** Hierarchical cluster tree diagram of co-expression modules based on a topological overlap matrix (1-TOM). Each branch in the clustering tree represents a gene, and co-expression modules are constructed and represented by different colors. **(C)** The heatmap of the co-expression network. **(D)** Correlation of the key modules and control and endometriosis group. Each row corresponds to a module eigengene, column to a clinical trait. Each module includes the respective correlation coefficient and corresponding *p*-value. And we identified turquoise and blue modules are correlated with the diseases.

Firstly, we integrated genes from GSE51981 dataset combined with ferroptosis genes from FerrDb dataset, and the four ferroptosis modules (Turquoise, Blue, Brown, and Grey) related to EMs and controls were identified through hierarchical clustering analysis and a dynamic cutting algorithm using a cut-off R^2^ value of 0.93 and an appropriate soft threshold β value (**Figures 1A–C**). Among these modules, the turquoise module exhibited a significant positive correlation with EMs, having a correlation coefficient of 0.25, while the blue module showed a significant negative correlation with EMs, with a correlation coefficient of -0.33 (**Figure 1D**). Consequently, we focused on analyzing the turquoise and blue modules in subsequent investigations.

### 4.2 Enrichment analysis of key modules

**Figure 2:**
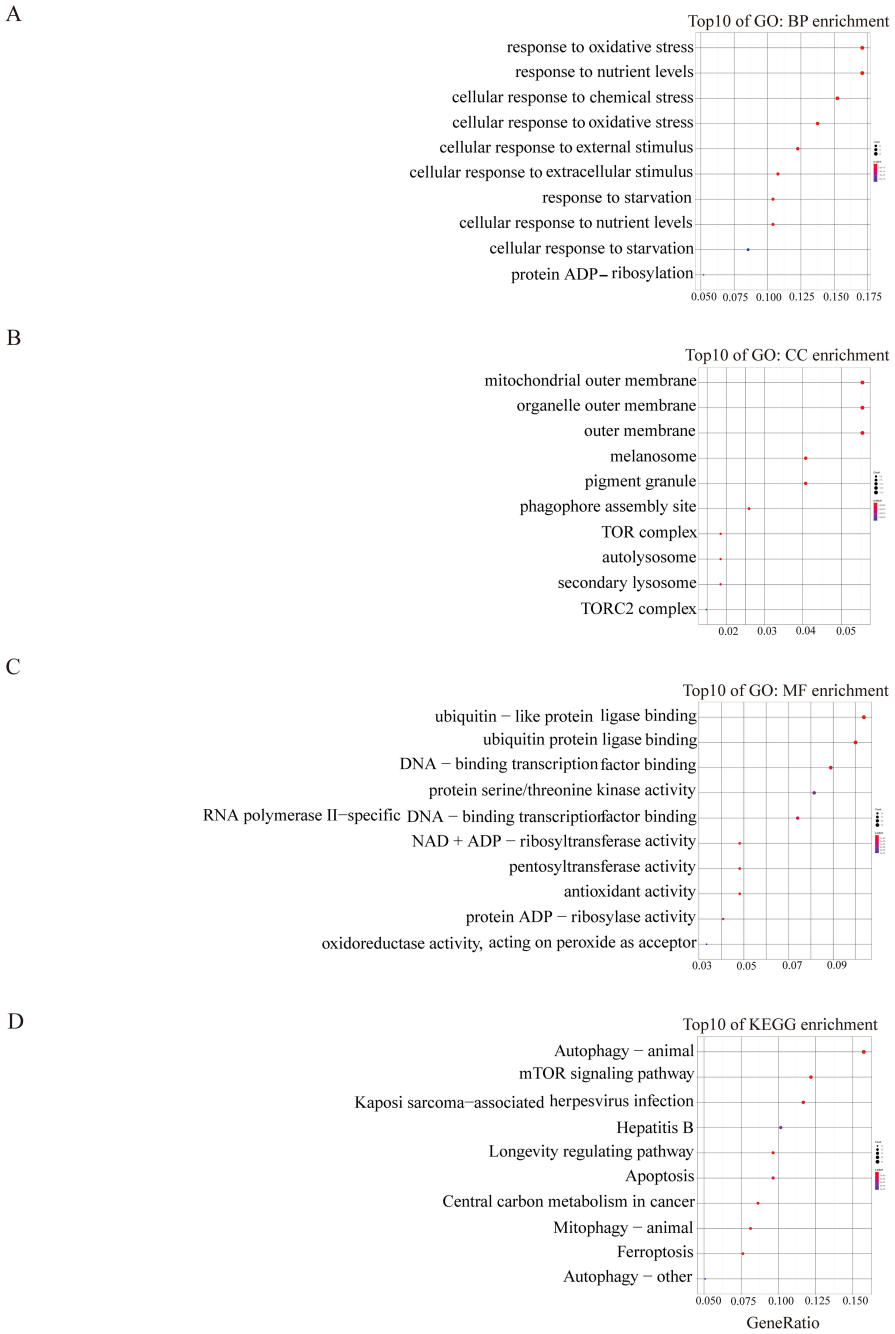
GO and KEGG enrichment analysis of turquoise module **(A–D)**. The color and size of each circles represent p-value and the number of the enriched genes, respectively.

**Figure 3:**
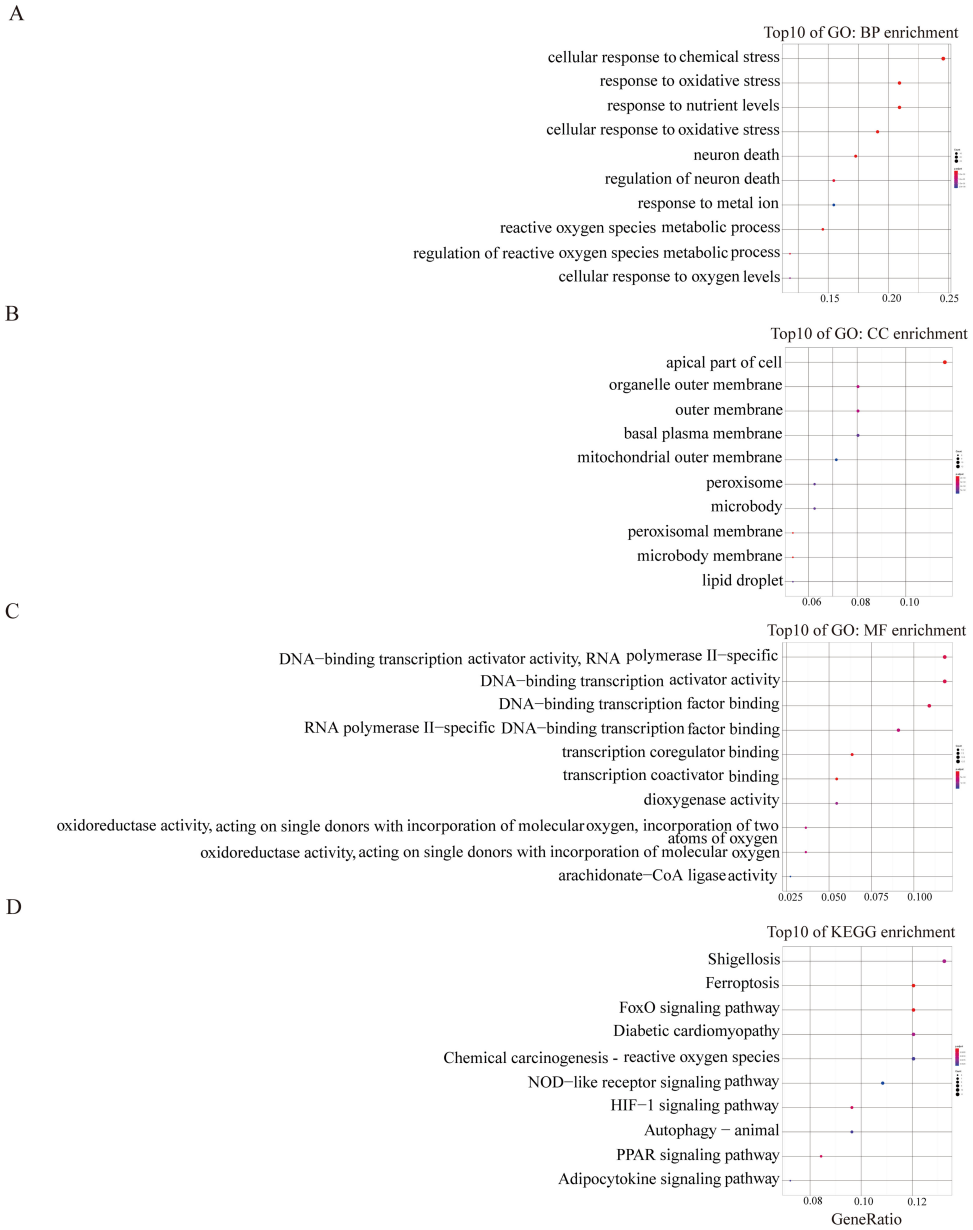
GO and KEGG enrichment analysis of blue module **(A–D)**. The color and size of each circles represent p-value and the number of the enriched genes, respectively.

Subsequent to this, we conducted GO and KEGG pathway analyses to elucidate the potential molecular functions and biological processes of the turquoise and blue module. The results depicted in **Figure 2** illustrated that the turquoise module predominantly correlated with oxidative stress, autophagy, and mTOR signalling pathways (**Figures 2A–D**). The blue module was primarily linked to shigellosis, ferroptosis, FOXO, and the NOD-like receptor signalling pathway (**Figures 3A–D**). These findings suggest a potential role of these pathways in modulating the ferroptosis process in EMs.

### 4.3 Identification of the hub FRGs

**Figure 4:**
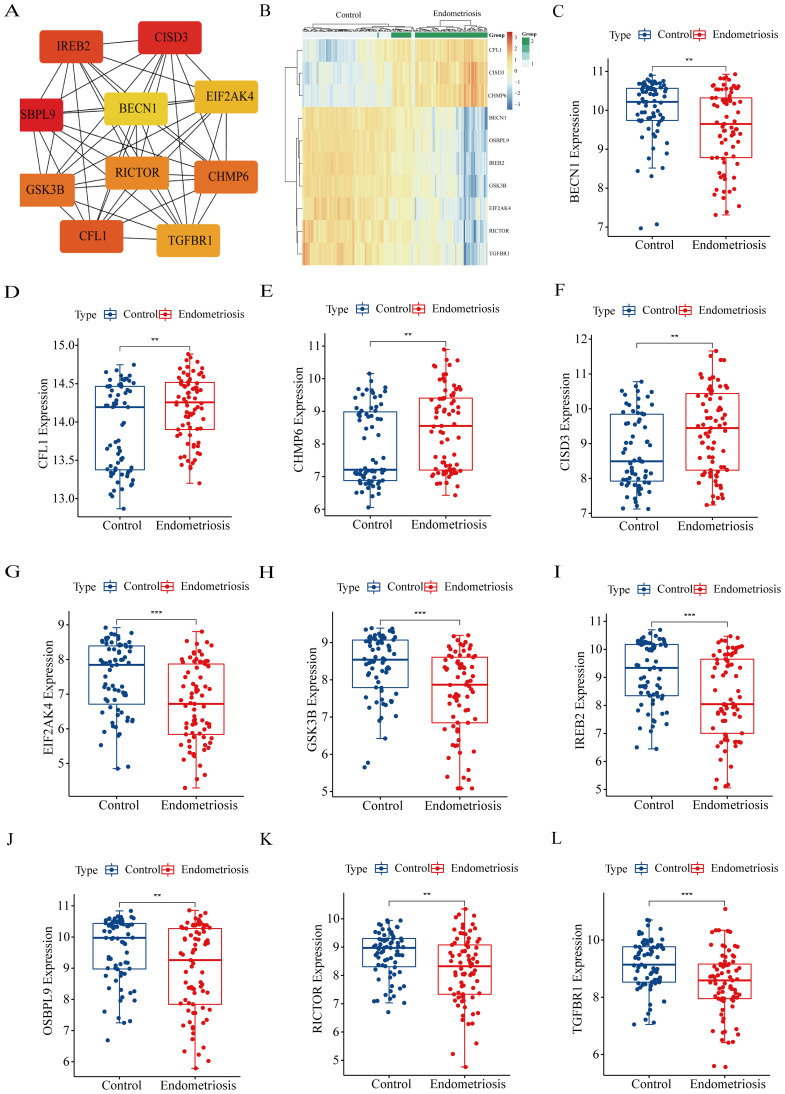
Identification and the expression levels of hub FRGs. **(A)** Identification of hub FRGs using MNC algorithm. Three indicators (degree, closeness and betweenness) were, respectively, calculated to evaluate the importance of each node and the top 10 nodes were selected. The six hub genes were their common nodes. **(B)** The heatmap of hub FRGs in GSE51981. **(C–L)** The expression box plots of hub FRGs in GSE51981. *P<0.05, **P<0.01, ***P<0.001.

It’s well known that the hub genes represent the biological significance of key modules, thus, we identified 10 hub FRGs in the aforementioned modules by the MNC algorithm (**Figure 4A**). Subsequently, we evaluated the expression level of hub FRGs in GSE51981 (**Figure 4B**). As shown in **Figures 4C–L**, CFL1, CHMP6, and CISD3 exhibited higher expression levels in the eutopic endometrium of patients with EMs compared to the normal endometrium of controls. Conversely, BECN1, EIF2AK4, GSK3B, IREB2, OSBPL9, RICTOR, and TGFBR1 were found to be upregulated in control patients when compared to those with EMs. Furthermore, we also validated the expression of hub FRGs in the GSE25628 database. The expression trends of most genes are consistent with those in GSE51981, which indicates that the genes we have screened are representative and reliable (**Supplementary Figure S1**).

### 4.4 Diagnostic values of hub FRGs

**Figure 5:**
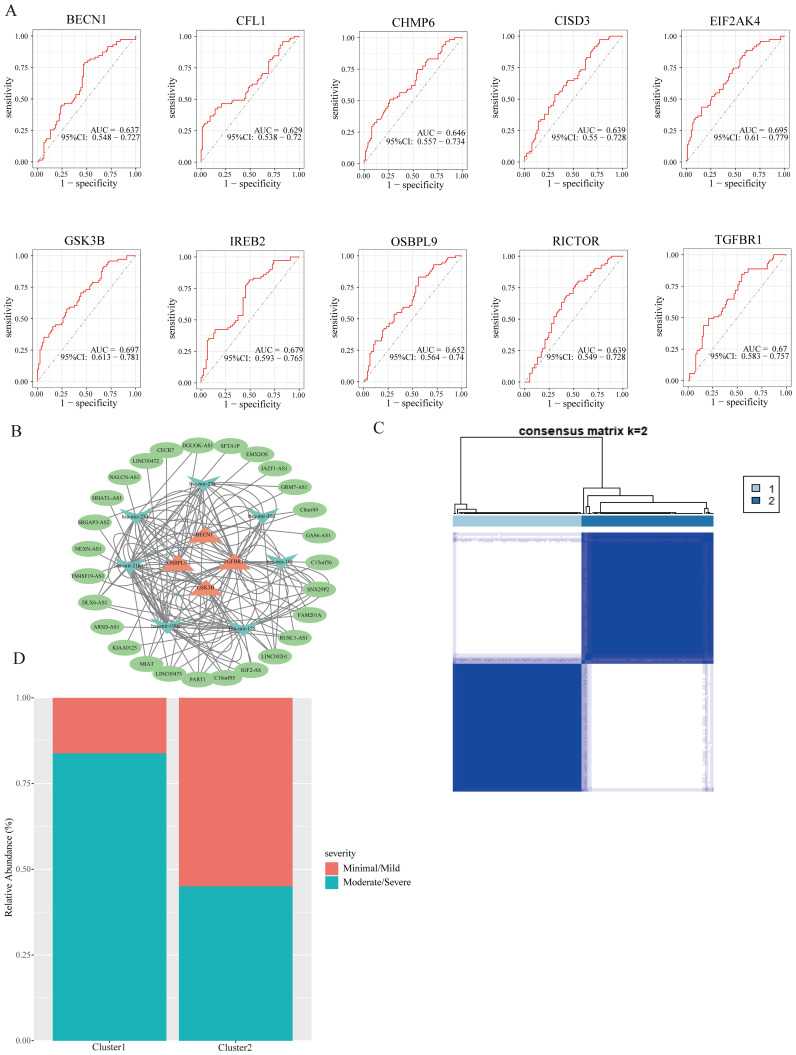
The ROC curve and ceRNA network of hub FRGs, and identification of subclusters. **(A)** The ROC curves of hub FRGs. **(B)** CeRNA network of hub FRGs. **(C)** Two ferroptosis subclusters were identified by consensus clustering analysis of EMs patients in GSE51981. **(D)** The relationship between EMs staging and subclusters.

To evaluate the diagnostic efficacy of hub FRGs, we conducted ROC curve analysis. The results depicted in **Figure 5A** showed that the area under the curve (AUC) of ROC for all 10 hub FRGs exceeded 0.6 in GSE51981. Specifically, the AUC values were 0.637 for BECN1, 0.629 for CFL1, 0.646 for CHMP6, 0.639 for CISD3, 0.695 for EIF2AK4, 0.697 for GSK3B, 0.679 for IREB2, 0.652 for OSBPL9, 0.639 for RICTOR, and 0.67 for TGFBR1. These results suggest their potential as diagnostic markers.

### 4.5 Construction of ceRNA network

Numerous evidence has been suggested that the ceRNA regulatory network plays a vital role in the pathological processes of diseases. Therefore, to further understand the regulatory mechanism of hub FRGs in EMs, we also constructed the ceRNA network using GSE105765 and GSE105764 combined with several bioinformatics database, which involves 4 hub FRGs (BECN1, TGFBR1, GSK3B, and OSBPL9), 7 miRNAs, and 27 lncRNAs (**Figure 5B**). These results provide new insights for further understanding the regulatory mechanisms of hub FRGs in EMs.

### 4.6 Identification of new ferroptosis subclusters in endometriosis

Although molecular subtypes have become crucial for predicting disease prognosis and guiding targeted therapy, there is currently no molecular subgroup identified for EMs. To address this gap, a cluster analysis was conducted to identify ferroptosis subclusters based on the FRGs in EMs samples from GSE51981. The analysis revealed that the most stable number of subclusters was achieved when k=2 (**Figure 5C**), which was further validated in GSE25628 databases (**Supplementary Figure S1**). Subsequent evaluation of the distribution of EMs stages in the two ferroptosis subclusters showed that cluster 1 was predominantly associated with moderate/severe stages of EMs, while cluster 2 had a higher abundance of minimal/mild stages compared to moderate/severe stages (**Figure 5D**). This suggests that the two ferroptosis subclusters could reflect to some extent the severity of conditions in EMs and are worth further investigation.

### 4.7 Immune landscapes of ferroptosis subclusters

**Figure 6:**
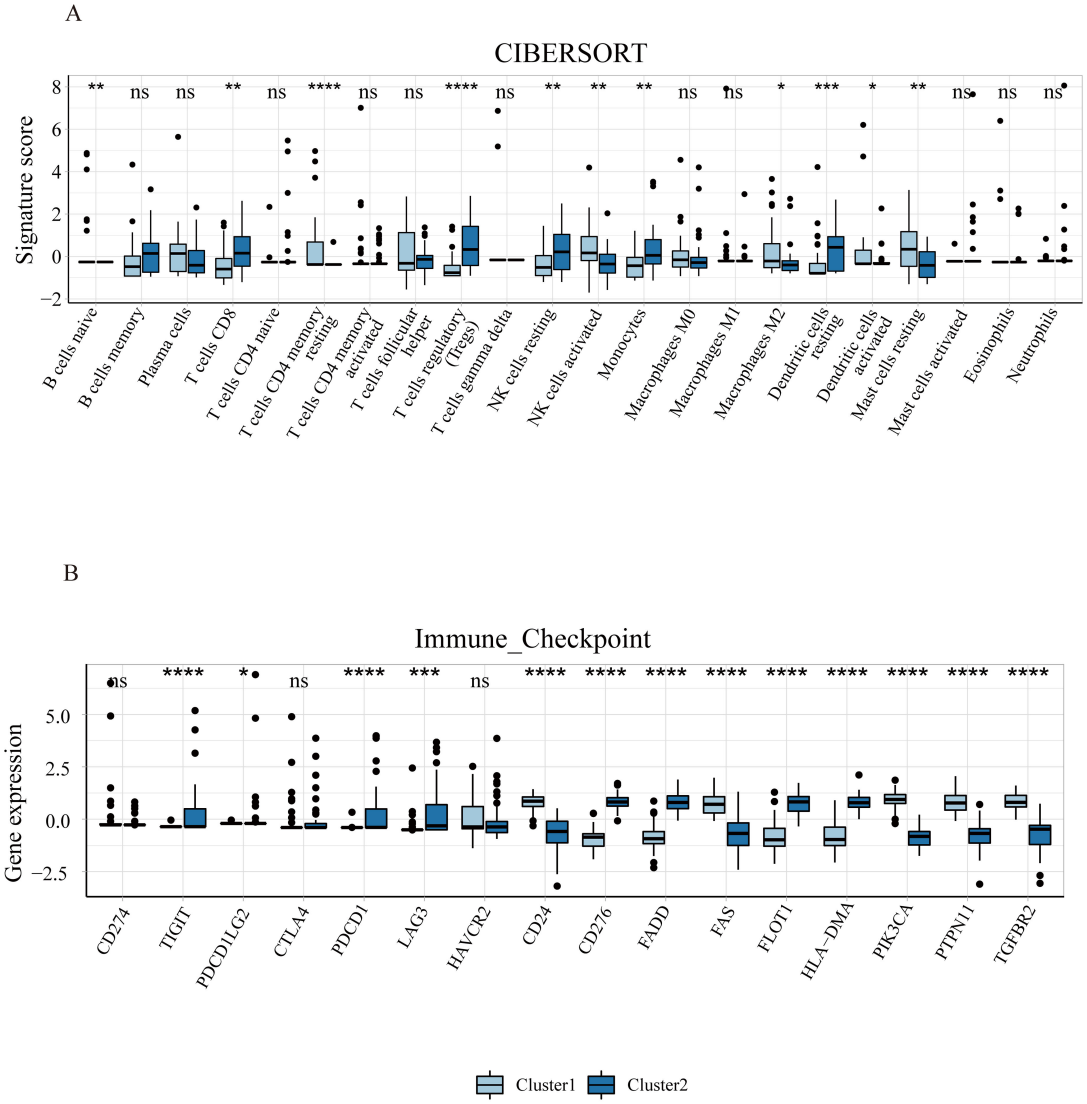
Immune infiltration of ferroptosis subclusters in endometriosis. **(A)** The difference of 22 types of immune cells’ infiltration between the two subclusters. **(B)** The expression levels of immune checkpoint genes in the two subclusters. *P<0.05, **P<0.01, ***P<0.001, ****P<0.0001.

Emerging evidence suggests a close relationship between ferroptosis and immunity (25, 26). Therefore, we analyzed the immune landscapes of two subclusters using CIBERSORT (**Figure 6**). In cluster 1, there was a higher proportion of B cells naïve, T cells CD4 memory resting, activated NK cells, M2 macrophages, activated dendritic cells, and resting mast cells, while cluster 2 showed a higher proportion of T cells CD8, regulatory T cells, resting NK cells, monocytes, and resting dendritic cells. Furthermore, we also examined the expression of immune checkpoint genes in these subclusters and found statistically significant differences in most immune checkpoint genes. Our results depict the landscape of ferroptosis and immune microenvironment of EMs interacting closely, providing a theoretical basis for further research on the specific mechanisms of the two in the disease.

### 4.8 Tissue expression of hub FRGs and its relationship with clinicopathological features

**Figure 7:**
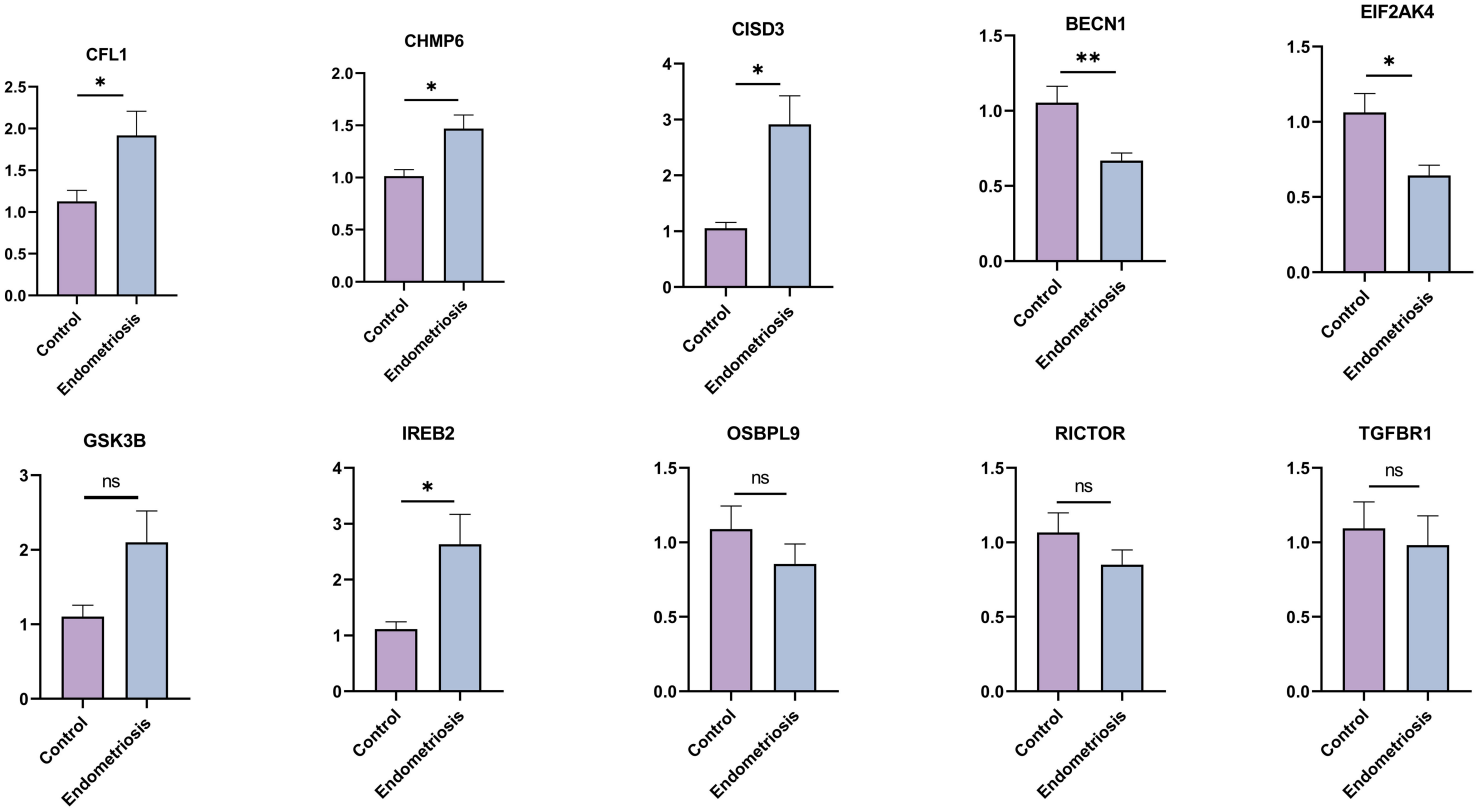
The expression levels of hub FRGs in tissue samples by RT-qPCR. Control group: normal endometrium of CINIII patients (n=10); Endometriosis group: eutopic endometrium of endometriosis patients (n=22). The data are presented as the Mean ± SD. *P<0.05, **P<0.01.

To further verify our results, we assessed the expression levels of 10 hub FRGs in the eutopic endometrium of endometriosis patients (Endometriosis group) and in the normal endometrium of patients with CIN III (Control group) (**Figure 7**). As we can see, the expression trend of hub FRGs in clinical samples was roughly consistent with the results of GSE51981, which confirms the reliability of our results. Furthermore, based on the median value of each hub FRGs expression, we analyzed the correlation between the expression of these hub FRGs and the clinicopathological features of endometriosis patients (**Supplementary Tables S1**-**S6**). Specifically, the expression of BECN1 positively correlates with disease staging and negatively correlates with CA125 levels. The expression of CFL1 is associated with a history of infertility. The expressions of CISD3 and CHMP6 negatively correlate with disease staging. The expression of IREB2 positively correlates with CA125 levels and the diameter of ovarian cysts, and negatively correlates with disease staging.

## 5 Discussion

The pathogenesis of EMs remains unclear. Although some studies suggest a potential involvement of ferroptosis in the development of EMs, its precise role remains incompletely understood. In this study, we utilized the latest ferroptosis-related genes retrieved from the FerrDb database and employed the WGCNA to identify ferroptosis modules that are most relevant to EMs. Subsequently, functional enrichment analysis demonstrated that the turquoise module, positively correlated with EMs, was primarily associated with oxidative stress, autophagy, and the mTOR signaling pathway. Conversely, the blue module, which demonstrates a negative correlation with EMs, was predominantly implicated in shigellosis and ferroptosis, while also being associated with the FOXO and NOD-like receptor signaling pathways. Furthermore, existing evidence supports the association of these pathways with the pathological process of EMs. Autophagy is a crucial cellular degradation pathway responsible for eliminating damaged or aged organelles (27). Research has shown that in normal endometrial cells, the induction of autophagy exhibits a pro-apoptotic effect, whereas the autophagy pathway is suppressed in ectopic endometrial cells of EMs patients (28). A recent study has revealed that, in EMs, Açai Berries promotes autophagy by reducing mTOR expression to regulate the PI3K/AKT/ERK1/2 pathway. Meanwhile, it also activates mitochondrial autophagy, which enhances the clearance of damaged mitochondria, reduces ROS production, and restores oxidative balance (29). Furthermore, it has been reported that mTOR, an important negative regulator of autophagy, is abnormally activated in the endometriotic lesions (30). The FOXO and NOD-like receptor signaling pathway participate in the inflammation process and cytokine signal transduction of EMs (31, 32). Consequently, these pathways might play a pivotal role in the involvement of ferroptosis in the development of EMs.

Next, we identified 10 hub FRGs using the MNC algorithm, including BECN1, GSK3B, IREB2, OSBPL9, EIF2AK4, RICTOR, TGFBR1, CFL1, CHMP6, and CISD3, which are potentially involved in the development of EMs through bioinformatics analysis. The AUC-ROC of all hub FRGs exceeded 0.6, indicating the high sensitivity and specificity of these hub FRGs in distinguishing EMs patients from healthy controls. Among these genes, BECN1, GSK3B, IREB2, OSBPL9, EIF2AK4, RICTOR, and TGFBR1 were downregulated in the eutopic endometria of EMs patients compared to the endometria of normal controls. In contrast, CFL1, CHMP6, and CISD3 were upregulated in EMs patients. Beclin1 (BECN1), a key component of the class III phosphatidylinositol 3-kinase (PtdIns3K) complex, is crucial in regulating macroautophagy/autophagy (33, 34). It inhibits system Xc− by interacting with SLC7A11, consequently promoting ferroptosis (35, 36). Meanwhile, hucMSC-exosome-derived BECN1 enhances HSC ferroptosis by inhibiting the expression of cystine/glutamate transporter (xCT)-driven GPX4 (37). Previous studies have shown that BECN1 expression was decreased in both eutopic and ectopic endometrium of patients with EMs compared to normal endometrium (38, 39), which is consistent with our bioinformatics analysis and PCR results. BECN1 plays a role in processes such as progesterone synthesis and apoptosis in EMs through its involvement in autophagy (40, 41). However, its role in ferroptosis in EMs remains unclear, and we will investigate this in subsequent experiments. Iron-responsive element binding protein 2 (IREB2) is a pivotal posttranscriptional regulator of cellular and systemic iron metabolism. A recent study found that miR-19a targets IREB2 to inhibit ferroptosis in colorectal cancer (42). Additionally, the upregulation of IREB2 disrupts iron homeostasis, leading to the accumulation of a labile iron pool and lipid peroxidation, which in turn triggers ferroptosis (43, 44). Previous research has indicated that iron overload and lipid peroxidation are implicated in the pathogenesis of EMs. Our results indicated that IREB2 was downregulated in the eutopic endometrium of EMs patients. Thus, based on the existing evidence, we hypothesize that IREB2 may play a crucial role in regulating ferroptosis through its influence on iron balance in endometrial cells, warranting additional study. GSK3B, a widely expressed serine/threonine protein kinase, is a key regulator of redox homeostasis by modulating nuclear factor erythroid 2-related factor (Nrf2) (45, 46). Wu et al. demonstrated that GSK3B inhibited Nrf2 expression, leading to the accumulation of ROS and malondialdehyde, thereby promoting erastin-induced ferroptosis in breast cancer cells (47). Our study indicated that GSK3B was downregulated in eutopic endometrium compared to normal endometrium, suggesting that GSK3B may play a role in suppressing ferroptosis in eutopic endometrium through similar mechanisms mentioned above. Transforming growth factor beta receptor 1 (TGFBR1, also known as ALK4/5) plays a crucial role in transmitting extracellular stimuli to the downstream TGF-β signalling pathway (48). Research has demonstrated that the inhibition of ALK4/5 effectively attenuates erastin-induced ferroptosis in HK-2 cells by potentiating Nrf2 signalling pathways (49) Previous studies have indicated that TGFBR1 expression was elevated in ectopic endometrium compared to eutopic endometrium in EMs patients (50). However, our research demonstrated that TGFBR1 was upregulated in normal endometrium compared to eutopic endometrium. This inconsistency could be attributed to variations in the tissue samples utilized in the two studies. Although TGFBR1 is involved in the migration and invasion processes of EMs, its role in ferroptosis within this condition remains elusive and deserves further exploration. Eukaryotic translation initiation factor 2 alpha kinase 4 (EIF2AK4, also known as GCN2) primarily participates in the cellular amino acid starvation response and regulates the immune response (51). The activation of the GCN2/ATF4 axis is triggered under cystine-deprived or xCT-KO conditions, thus contributing to ferroptosis (52, 53). Oxysterol-binding protein-like 9 (OSBPL9), a member of the oxysterol-binding protein family, is localized at the cellular membrane and exchanges molecules or signals between organelles, functioning as an essential transporter (54). Although there are few studies on the relationship between OSBPL9 and ferroptosis, OSBPL9 may be involved in ferroptosis by regulating the integration of sterol and sphingomyelin metabolism, sterol transport, and neutral lipid metabolism (55). Rapamycin-insensitive companion of mTOR (RICTOR) is a core component and functional element of mTORC2 that participates in PI3K/AKT pathway activation, cell proliferation, migration, autophagy, and metabolism (56). Research demonstrated that mTORC2 inhibited xCT activity through serine 26 phosphorylation at the cytosolic N-terminus (57), which may trigger ferroptosis. However, further investigations are warranted to elucidate the relationship between RICTOR and ferroptosis. Charged multivesicular body protein 6 (CHMP6) is an ESCRT-III subunit that is involved in membrane repair to inhibit necroptosis and pyroptosis (58). Dai et al. discovered that the silencing of CHMP6 expression inhibits the repair of the ferroptotic plasma membrane and enhances ferroptosis by modulating lipid peroxidation and DAMP release (59). CDGSH iron sulfur domain 3 (CISD3), also known as Miner2 or MiNT, is a mitochondrial protein belonging to the NEET protein family. Members of the NEET family are key regulators of iron and ROS homeostasis (60). CISD3 downregulation leads to a reduction in the mitochondrial membrane potential (MMP) and an increase in mitochondrial labile iron and ROS accumulation, thereby triggering ferroptosis (61). Cofilin-1 (CFL1) is a member of the ADF/cofilin family and plays a crucial role in actin depolymerization (62). Existing evidence suggests that the upregulation of CFL1 increases glutamate- and elastin-induced ferroptosis (63). Moreover, CFL1 triggers ferroptosis by modulating NF-κB signalling or the ER stress pathway to induce acute kidney injury (AKI) (64). Prior study suggested that CFL1 was significantly upregulated in eutopic endometrium from EMs patients compared to the normal endometrium (65), which is consistent with our results. The high expression of CFL1 in eutopic endometrium can promote the proliferation, adhesion, and invasion of endometrial stromal cells (ESC), while inhibiting apoptosis. However, there is no research on the role of CFL1 in ferroptosis in EMs. Taken together, known evidence from prior research, along with our findings, demonstrates that further elucidating the role of these hub FRGs in ferroptosis in EMs may provide potential therapeutic targets for this disorder. Currently, targeting ferroptosis seem to a promising therapy for treating diseases (66). However, given the dual role of ferroptosis in EMs and its complex interaction with the immune system, it is uncertain which targeted ferroptosis treatment methods, such as inhibiting ferroptosis or promoting ferroptosis, have a definitive therapeutic effect on EMs. This needs further research in the future to confirm.

It is reported that a large number of miRNAs and lncRNAs are involved in the occurrence and development of EMs. However, the current research focusing on the correlation between non-coding RNA and ferroptosis has primarily concentrated on cancer, with comparatively fewer reports in the context of endometriosis. Herein, we have constructed a ceRNA network of hub FRGs, which includes 4 hub FRGs, 7 miRNAs, and 27 lncRNAs. These miRNAs are closely related to EMs or ferroptosis, such as miR-119a, miR-128, miR-101, miR-30a, miR-27a, miR-223, and miR-216a. For example, miR-119a is downregulated in the ectopic stromal cells (ESCs) of patients with ovarian EMs which inhibits the migration and invasion of ESCs by targeting PAK4 (67). miR-223 inhibits the proliferation, migration and invasion and promotes apoptosis of ESCs in EMs. In hepatitis B-related viral nephritis, exosomal miR-223-3p derived from bone marrow mesenchymal stem cells downregulates STAT3 phosphorylation by targeting HDAC2, thereby alleviating hbx-induced ferroptosis in renal podocytes (68). Meanwhile, we have also identified 27 lncRNAs, which can participate in the progression of multiple diseases through mediating various mechanisms. In summary, the ceRNA network of hub FRGs constructed in our study provides new insights for further understanding the regulatory mechanisms of ferroptosis in EMs.

Various diseases have recently been classified into molecular subtypes to guide prognosis and targeted treatment. In this study, we identified two ferroptosis subclusters related to EMs based on FRGs. Among them, Cluster 1 was mainly associated with moderate/severe stages, while Cluster 2 was predominantly linked to minimal/mild stages. Previous research has shown a strong connection between ferroptosis and immunity, yet a comprehensive analysis of the relationship between ferroptosis and immune infiltration in EMs remains unexplored. Using CIBERSORT, we determined the proportions of different immune cell types in the two clusters. Interestingly, Cluster 1 showed higher proportions of immune cells like activated dendritic cells (DCs), activated natural killer (NK) cells, and M2 macrophages, while Cluster 2 had elevated levels of immunosuppressive cells such as regulatory T cells (Tregs). Macrophages facilitate the progression of EMs by attenuating their phagocytic capacity and promoting the synthesis of inflammatory mediators, while M2 macrophages are involved in anti-inflammatory processes, immunosuppression, and neuroangiogenesis (67). Dendritic cells (DCs) act as a bridge between innate and adaptive immune responses. Prior studies revealed a greater proportion of immature DCs (iDCs) in the peritoneal DCs of EMs patients than in those of control patients, while iDCs facilitate angiogenesis, immune tolerance and the establishment of endometriotic foci (69). NK cells, cytotoxic effector lymphocytes, can regulate innate and adaptive immune responses. Wu et al. demonstrated that activated NK cells were more abundant in the eutopic endometria of patients with EMs than in those of controls (70), which is in accordance with our results. Tregs are a specialized subset of T cells primarily generated in the thymus that exert suppressive effects on immune responses. They are more concentrated within the peritoneal fluid of EMs and facilitate local immunosuppression and induce angiogenesis by interacting with proinflammatory cytokines (71). These results seemingly suggest that stage III-IV exhibits greater immunosuppressive and angiogenic capacity than stage I-II thus promoting the growth of endometriotic lesions, which is consistent with the distribution of immune cells in disease severity in our results. Moreover, immune checkpoint genes were notably differentially expressed between the two clusters. Specifically, TIGIT, PDCD1LG2, PDCD1, LAG3, CD276, FADD, FLOT1 and HLA-DMA are notably high expressed in cluster 2. While the CD24, FAS, PIK3CA, PTPN11 and TGFBR2 are significantly upregulated in cluster 1. These genes play an immunosuppression role in the progression of diseases. Such as, TIGIT, identified in 2009, is expressed on Tregs, memory T cell subsets, and NK cells which induces the exhaustion of immune cells and mediates immune suppression (72). PDCD1, also known as PD-1, is a vital immune checkpoint gene for cancer immunotherapy, which interactions with PD-L1 negatively regulating adaptive immune response mainly by inhibiting the activity of effector T cells and enhancing the function of immunosuppressive Tregs (73). Previous studies demonstrated a notable upregulation of PD-1 and PD-L1 expression in EMs (74, 75), suggesting that targeting PD-1 maybe a promising immunotherapy for EMs. LAG3, often expresses on the lymphocytes, including CD4^+^ T cells, NK cells, CD8^+^ T cells, and Treg cells, impeding the tumor immune microenvironment through accelerating T cell exhaustion and blocking T cell proliferation. As a result, high LAG3 expression promotes tumor growth by inhibiting the immune microenvironment (76). CD276 (B7-H3), a novel immune checkpoint of the B7 family, is widely overexpressed in tumor tissues associated with a worse prognosis and provides an immunosuppression environment for diseases progression (77). CD24 is predominantly expressed on the surface of the T and B lymphocytes and is overexpressed in multiple cancer cell types. During the interaction between immune cells and cancer cells, it inhibits phagocytosis leading to tumor-mediated immune escape (78). Fas participates in the regulation of cell death, plays a key role in immune homeostasis and immune surveillance (79). PIK3CA, a most frequently mutated gene, could activate multiple genetic programs and influence intratumoral heterogeneity which its wild types may have favorable immunotherapy outcomes for patients with breast cancer (80). In a word, our findings suggest that these immune checkpoint genes may provide a favorable immune environment for the progression of EMs. However, we must admit that, although our study indicates that these genes may play a role in the development of different stages of EMs, there are currently limited reports on their specific functions in EMs thus whether these genes are indeed associated with the stage of the disease still requires further confirmation through future research. These findings suggest that ferroptosis is closely related to immune microenvironment during the progression of EMs and further provides the new insights for understanding the interaction between the both.

There are several limitations that must be noted. First, further functional experiments are warranted to verify the role and mechanisms of the hub FRGs in EMs. Second, *in vitro* and *in vivo* experiments are needed to confirm the interaction between ferroptosis and immune cells in EMs. Subsequently, we will undertake additional experiments to confirm the findings of this study.

## 6 Conclusion

In summary, our study has revealed that autophagy, mTOR, oxidative stress, and FOXO signaling pathway may play a crucial role in the process of ferroptosis in EMs and provided the evidence that ferroptosis could be involved in reshaping the immune microenvironment in EMs. Additionally, we have also identified 10 key FRGs and constructed their regulatory network, which will enhance our understanding of the role of ferroptosis in EMs. Taken together, our study provides valuable information for understanding ferroptosis and immunity of EMs, and lays a theoretical foundation for subsequent research.

## Data Availability

The original contributions presented in the study are included in the article **Supplementary Material**. Further inquiries can be directed to the corresponding author.

## Supplementary material

Supplementary Figure 1 The expression levels of hub FRGs in the GSE25628 databases. **(A–G)** The expression box plots of hub FRGs in GSE25628. **(F)** Two ferroptosis subclusters were identified by consensus clustering analysis of EMs patients in GSE25628.

Supplementary Table 1 The correlation of the expression of BECN1 and clinicopathological features in endometriosis patients.

Supplementary Table 2 The correlation of the expression of EIF2AK4 and clinicopathological features in endometriosis patients.

Supplementary Table 3 The correlation of the expression of CFL1 and clinicopathological features in endometriosis patients.

Supplementary Table 4 The correlation of the expression of CISD3 and clinicopathological features in endometriosis patients.

Supplementary Table 5 The correlation of the expression of CHMP6 and clinicopathological features in endometriosis patients.

Supplementary Table 6 The correlation of the expression of IREB2 and clinicopathological features in endometriosis patients.
