# Supplementary figures and images for: New insights into the ferroptosis and immune infiltration in endometriosis: a bioinformatics-based analysis
Source: Front Immunol. 2025 Jan 13;15:1507083. doi: 10.3389/fimmu.2024.1507083 (PMC11769811; doi:10.3389/fimmu.2024.1507083)

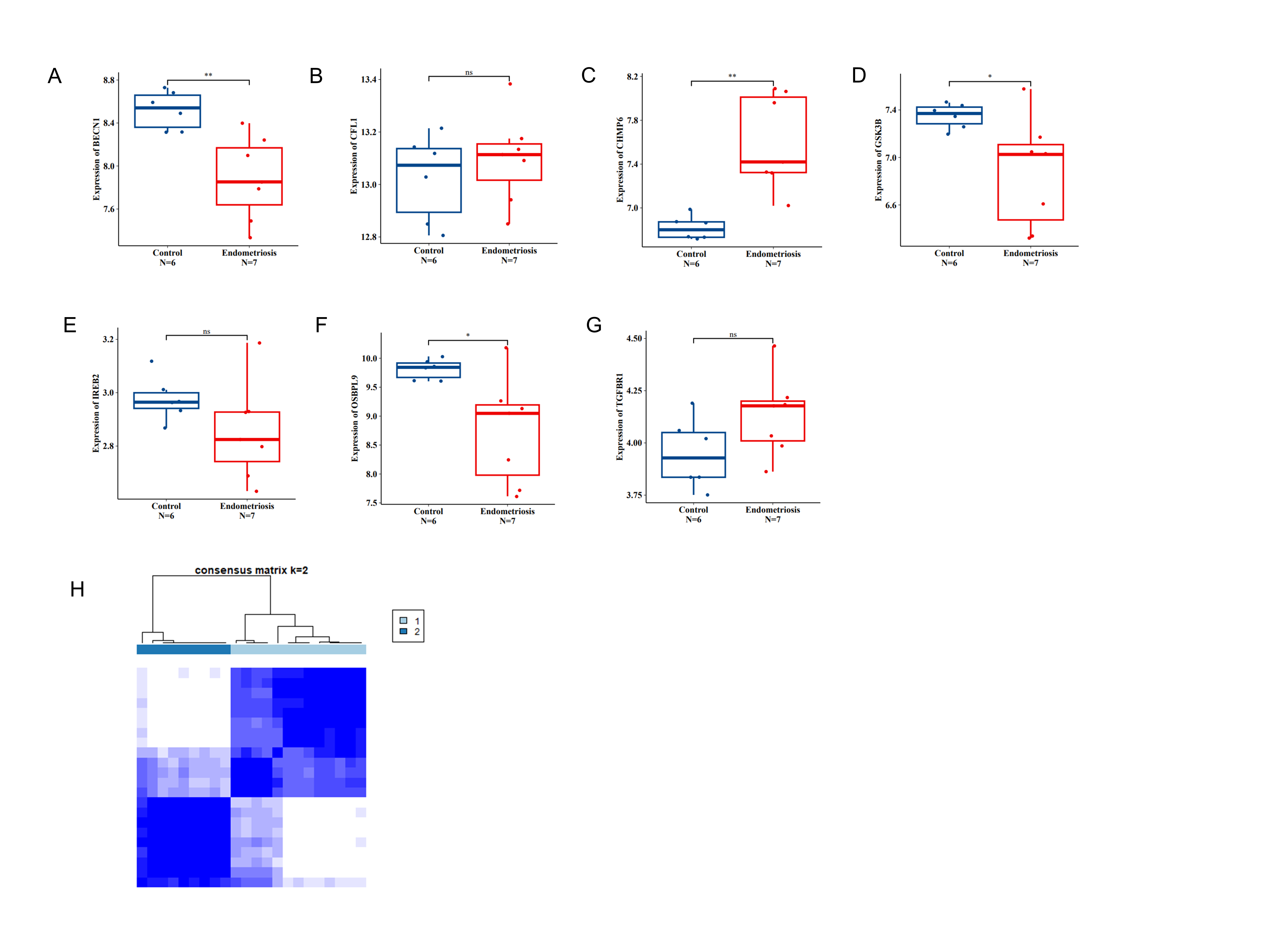

Supplement: Supplementary Figure 1 — The expression levels of hub FRGs in the GSE25628 databases. (A–G) The expression box plots of hub FRGs in GSE25628. (F) Two ferroptosis subclusters were identified by consensus clustering analysis of EMs patients in GSE25628. [file Image1.tif]
