# Supplementary material for: New insights into the ferroptosis and immune infiltration in endometriosis: a bioinformatics-based analysis
Source: Front Immunol. 2025 Jan 13;15:1507083. doi: 10.3389/fimmu.2024.1507083 (PMC11769811; doi:10.3389/fimmu.2024.1507083)
Supplement: Supplementary Table 1 — The correlation of the expression of BECN1 and clinicopathological features in endometriosis patients. [file DataSheet1.zip › supplementary Table S1-S6/Table S1-6.docx]

| clinicopathological features | BECN1 | | *P*-value |
| --- | --- | --- | --- |
|  | Low expression（n=11） | High expression（n=11） |  |
| age | 37.64±6.93 | 36.91±6.92 | 0.808 |
| history of infertility |  |  | 0.611 |
| No | 9（81.8%） | 8（72.7%） |  |
| Yes | 2（18.2%） | 3（27.3%） |  |
| unilateral and bilateral side |  |  | 0.375 |
| unilateral side | 6（54.5%） | 5（45.5%） |  |
| bilateral sides | 8（72.7%） | 3（27.3%） |  |
| diameter of ovarian cyst | 6.13±1.91 | 5.17±1.77 | 0.235 |
| staging |  |  | **0.033** |
| rAFS I-II | 3（27.3%） | 8（72.7%） |  |
| rAFS III-IV | 8（72.7%） | 3（27.3%） |  |
| CA125 (U / mL) | 109.59±61.80 | 63.76±37.57 | **0.048** |

| clinicopathological features | EIF2AK4 | | *P*-value |
| --- | --- | --- | --- |
|  | Low expression（n=11） | High expression（n=11） |  |
| age | 37.55±6.61 | 37.00±7.24 | 0.855 |
| history of infertility |  |  | 0.611 |
| No | 8（72.7%） | 9（81.8%） |  |
| Yes | 3（27.3%） | 2（18.2%） |  |
| unilateral and bilateral side |  |  | 0.375 |
| unilateral side | 8（72.7%） | 6（54.5%） |  |
| bilateral sides | 3（27.3%） | 5（45.5%） |  |
| diameter of ovarian cyst | 5.12±1.71 | 6.19±1.93 | 0.184 |
| staging |  |  | 0.670 |
| rAFS I-II | 5（45.5%） | 6（54.5%） |  |
| rAFS III-IV | 6（54.5%） | 5（45.5%） |  |
| CA125 (U / mL) | 83.26±49.05 | 90.10±62.88 | 0.779 |

| clinicopathological features | CFL1 | | *P*-value |
| --- | --- | --- | --- |
|  | Low expression（n=13） | High expression（n=9） |  |
| age | 36.08±6.02 | 39.00±7.76 | 0.331 |
| history of infertility |  |  | **0.002** |
| No | 13（100%） | 4（44.4%） |  |
| Yes | 0（0） | 5（55.6%） |  |
| unilateral and bilateral side |  |  | 0.512 |
| unilateral side | 9（69.2%） | 5（55.6%） |  |
| bilateral sides | 4（30.8%） | 4（44.4%） |  |
| diameter of ovarian cyst | 5.70±1.49 | 5.59±2.41 | 0.895 |
| staging |  |  | 0.193 |
| rAFS I-II | 8（61.5%） | 3（33.3%） |  |
| rAFS III-IV | 5（38.5%） | 6（66.7%） |  |
| CA125 (U / mL) | 84.83±64.25 | 89.35±42.16 | 0.855 |

| clinicopathological features | CISD3 | | *P*-value |
| --- | --- | --- | --- |
|  | Low expression（n=11） | High expression（n=11） |  |
| age | 37.64±7.09 | 36.91±6.76 | 0.808 |
| history of infertility |  |  | 0.611 |
| No | 8（72.7%） | 9（81.8%） |  |
| Yes | 3（27.3%） | 2（18.2%） |  |
| unilateral and bilateral side |  |  | 0.076 |
| unilateral side | 9（81.8%） | 5（45.5%） |  |
| bilateral sides | 2（18.2%） | 6（54.5%） |  |
| diameter of ovarian cyst | 5.51±1.81 | 5.80±1.99 | 0.724 |
| staging |  |  | **0.003** |
| rAFS I-II | 9（81.8%） | 2（18.2%） |  |
| rAFS III-IV | 2（18.2%） | 9（81.8%） |  |
| CA125 (U / mL) | 78.78±37.52 | 94.58±69.58 | 0.517 |

| clinicopathological features | CHMP6 | | *P*-value |
| --- | --- | --- | --- |
|  | Low expression（n=12） | High expression（n=10） |  |
| age | 35.92±7.57 | 38.90±5.61 | 0.315 |
| history of infertility |  |  | 0.781 |
| No | 9（75.0%） | 8（80.0%） |  |
| Yes | 3（25.0%） | 2（20.0%） |  |
| unilateral and bilateral side |  |  | 0.035 |
| unilateral side | 10（83.3%） | 4（40.0%） |  |
| bilateral sides | 2（16.7%） | 6（60.0%） |  |
| diameter of ovarian cyst | 5.48±1.32 | 5.86±2.43 | 0.649 |
| staging |  |  | **0.010** |
| rAFS I-II | 9（75.0%） | 2（20.0%） |  |
| rAFS III-IV | 3（25.0%） | 8（80.0%） |  |
| CA125 (U / mL) | 80.66±40.20 | 93.90±70.81 | 0.588 |

| clinicopathological features | IREB2 | | *P*-value |
| --- | --- | --- | --- |
|  | Low expression（n=11） | High expression（n=11） |  |
| age | 35.18±5.51 | 39.36±7.50 | 0.152 |
| history of infertility |  |  | 0.127 |
| No | 10（90.9%） | 7（63.6%） |  |
| Yes | 1（9.1%） | 4（36.4%） |  |
| unilateral and bilateral side |  |  | 0.076 |
| unilateral side | 9（81.8%） | 5（45.5%） |  |
| bilateral sides | 2（18.2%） | 6（54.5%） |  |
| diameter of ovarian cyst | 4.74±1.56 | 6.57±1.74 | **0.017** |
| staging |  |  | **0.033** |
| rAFS I-II | 8（72.7%） | 3（27.3%） |  |
| rAFS III-IV | 3（27.3%） | 8（72.7%） |  |
| CA125 (U / mL) | 56.99±32.48 | 116.37±58.24 | **0.008** |
